# Nanoporous Carbons from Hydrothermally Treated Alga: Role in Batch and Continuous Capacitive Deionization (CDI)

**DOI:** 10.3390/molecules30132848

**Published:** 2025-07-03

**Authors:** Dipendu Saha, Ryan Schlosser, Lindsay Lapointe, Marisa L. Comroe, John Samohod, Elijah Whiting, David S. Young

**Affiliations:** Chemical and Materials Engineering Department, Widener University, 1 University Place, Chester, PA 19013, USAlmlapointe@widener.edu (L.L.); dsyoung@widener.edu (D.S.Y.)

**Keywords:** porous carbon, adsorption, capacitive deionization, water purification

## Abstract

This study presents a sustainable approach for synthesizing high-performance activated carbon from *Spirulina* Alga through hydrothermal carbonization followed by chemical activation using potassium hydroxide. The resulting activated carbon exhibited a high Brunauer–Emmett–Teller (BET) surface area of 1747 m^2^/g and a total pore volume of 1.147 cm^3^/g, with micropore volume accounting for 0.4 cm^3^/g. Characterization using Scanning Electron Microscopy-Energy Dispersive X-ray Spectroscopy (SEM-EDS), X-ray Photoelectron Spectroscopy (XPS), and gas adsorption analyses confirmed the presence of hierarchical micro- and mesoporosity as well as favorable surface functional groups. The synthesized carbon was used to fabricate electrodes for membrane capacitive deionization (MCDI) along with cation and anion-selective membranes, which were then tested with saline water (500–5000 ppm) and synthetic hard water (898 ppm of total salts). The salt adsorption capacity (SAC) reached 25 (batch) to 40 (continuous) mg/g, while rapid adsorption rates with average salt adsorption rates (ASARs) values exceeding 10 (batch) to 30 (continuous) mg·g^−1^·min^−1^ during early stages were obtained. Batch MCDI experiments demonstrated a higher SAC compared to continuous operation, with non-monotonic trends in SAC observed as a function of feed concentration. Ion adsorption kinetics were influenced by ion valency, membrane selectivity, and pore structure. The specific energy consumption (SEC) was calculated as 8–21 kJ/mol for batch and 0.1–0.5 kJ/mol for continuous process. These performance metrics are on par with or surpass those reported in the recent literature for similar single-electrode CDI configurations. The results demonstrate the viability of using Alga-derived carbon as an efficient and eco-friendly electrode material for water desalination technologies.

## 1. Introduction

Freshwater scarcity is one of the most pressing global challenges of the 21st century, intensified by rapid population growth, urbanization, industrial development, and climate change. According to the United Nations, nearly 2.2 billion people live in water-stressed countries, and this number is projected to rise significantly by 2050 [1]. In response to this escalating crisis, desalination technologies have emerged as viable solutions for augmenting freshwater supplies. However, conventional desalination methods, such as reverse osmosis (RO) [2,3], electrodialysis [4,5], and membrane filtration and extraction [6], are energy-intensive, capital-heavy, and often associated with environmental concerns, including brine disposal and chemical usage. These limitations have spurred global interest in alternative, energy-efficient desalination methods that are both cost-effective and environmentally friendly [7,8,9,10]. Among these, electrochemical and capacitive-based technologies have gained considerable traction for their lower energy consumption, modularity, and potential for integration with renewable energy sources [11,12,13,14].

**Figure 1:**
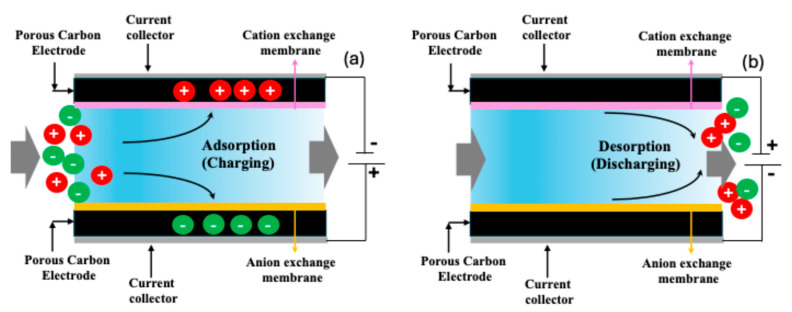
Schematic of membrane capacitive deionization (MCDI): (**a**) adsorption or charging step, where the ions from the feed water pass through the ion exchange membranes and are adsorbed in the carbon due to applied potential; (**b**) desorption or discharge where the adsorbed ions are released from the carbon due to altering the electrical potential.

Capacitive deionization (CDI) has emerged as a promising low-energy desalination technology suitable for treating low-to-moderate-salinity water. In CDI, ions are removed from water by applying a low electrical potential (typically < 1.5 V) across two porous electrodes, causing cations and anions to migrate and adsorb onto oppositely charged electrode surfaces. In order to regenerate the CDI system, the polarity of the electric potential to the electrodes is switched [11,12,15]. A schematic of CDI is shown in Figure 1. The process is cyclic and energy-efficient, with minimal chemical usage, making it attractive for decentralized and portable water purification systems. A key limitation of traditional CDI, however, is the co-ion expulsion effect, which can reduce the charge efficiency and salt adsorption capacity. To address this, membrane capacitive deionization (MCDI) introduces ion-exchange membranes in front of each electrode, significantly improving ion selectivity and preventing co-ion repulsion. This enhancement leads to higher salt removal efficiencies, better charge utilization, and a more stable cycling performance. MCDI thus represents a significant advancement over conventional CDI, especially for applications involving brackish water or hard water treatment, where selective ion removal is crucial [11,12,15].

The performance of CDI and MCDI systems is heavily influenced by the properties of the electrode materials. Porous carbon-based materials are particularly favored due to their excellent electrical conductivity, high specific surface area, chemical stability, and tunable pore structures. Among various carbon materials explored—such as activated carbon, carbon aerogels, carbon nanotubes, and graphene—activated carbon remains the most commercially viable and widely used electrode material for CDI due to its affordability and ease of processing [15]. More recently, there has been growing interest in developing activated carbons from renewable and sustainable biomass sources [16,17,18], driven by the need to reduce environmental footprints and promote circular economy principles. Biomass-derived carbons offer unique advantages, including natural heteroatom doping (e.g., nitrogen, oxygen) [19], hierarchical porosity, and low-cost feedstocks. Agricultural residues, food waste, and microalgae are increasingly being explored as precursor materials for high-performance carbon electrodes [20,21]. Among them, microalgae stand out due to their rapid growth rates, high carbon content, and the ability to sequester CO_2_, thus serving as an attractive carbon-negative feedstock for sustainable material synthesis.

In this study, we introduce an innovative strategy for synthesizing high-performance activated carbon from *Spirulina platensis*, a nutrient-rich microalgal biomass, specifically tailored for application in membrane capacitive deionization systems. While most previous works in this domain have focused on carbon materials derived from lignocellulosic biomass, agricultural residues, or food waste, our approach stands apart by utilizing *Spirulina*—a fast-growing and abundant microalga—as a sustainable and carbon-negative precursor. The carbonization and activation process employed in this work yields a porous carbon material with excellent electrochemical and structural properties conducive to efficient ion removal. Compared to the existing literature, this work offers significant novelty not only in the choice of biomass but also in the synthesis route, which combines hydrothermal carbonization with chemical activation to enhance surface properties and functionalization. The resulting electrodes demonstrate a robust desalination performance in both batch and continuous MCDI operations, positioning this study as taking a step forward in the development of next-generation desalination technologies. By integrating sustainable material sourcing with advanced electrochemical design, our work contributes a unique and environmentally conscious solution to the global water purification challenge.

## 2. Results and Discussion

### 2.1. Materials’ Characteristics

**Figure 2:**
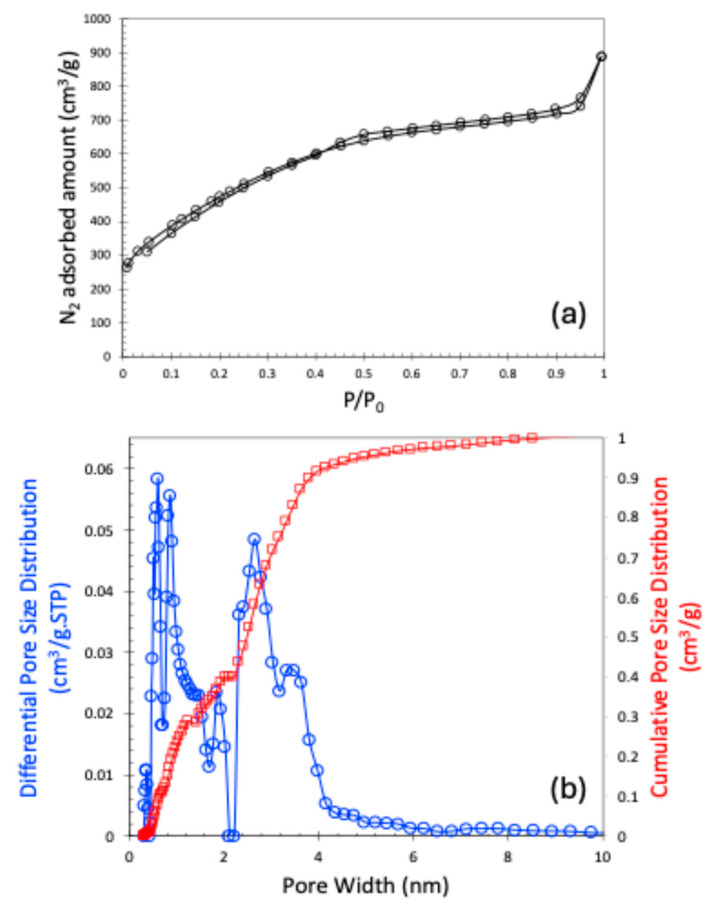
N_2_ adsorption–desorption plot at 77 K (**a**); NLDFT-based pore size distribution (**b**).

The nitrogen (N_2_) adsorption–desorption isotherm at 77 K is presented in Figure 2a. The observed isotherm closely resembles a Type I curve according to the International Union of Pure and Applied Chemistry (IUPAC) classification, indicating the presence of microporosity. Although the isotherm is primarily Type I, the subtle slope in the low relative pressure region suggests the presence of broader micropores. According to the IUPAC classifications, isotherms that exhibit a hysteresis loop are typically categorized as Type IV or Type V. In our case, however, the observed hysteresis loop is extremely narrow, which could be attributed to either a very limited amount of mesoporosity or potential experimental inconsistencies. For this reason, we classified the isotherm as Type I. Consequently, we also refrained from identifying the specific type of hysteresis present in the isotherm. This N_2_ adsorption–desorption data are used to calculate the Brunauer–Emmett–Teller (BET) surface area and the pore size distribution using the Non-Local Density Functional Theory (NLDFT). The sample exhibited a BET surface area of 1747 m^2^/g, a pore volume of 1.147 cm^3^/g, and a micropore volume of 0.4 cm^3^/g. The pore size distribution of the carbon material is shown in Figure 2b. It is noteworthy that pores smaller than 10 Å were analyzed using carbon dioxide (CO_2_) adsorption at 273 K, while the complete pore size distribution spectrum was obtained by combining data from both N_2_ and CO_2_ adsorption isotherms. As shown in Figure 2b, the carbon material primarily contains narrow micropores around 0.6 and 0.85 nm, and mesopores of 2.64 nm.

**Figure 3:**
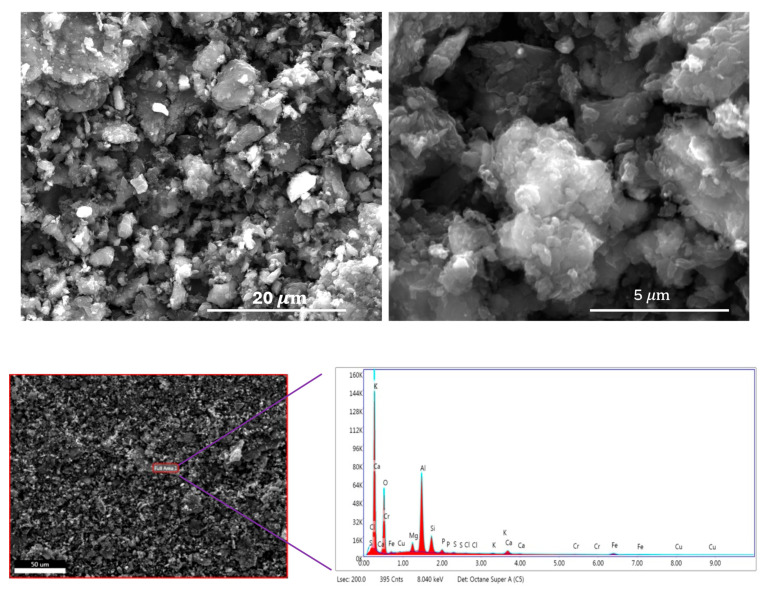
Scanning Electron Microscopic (SEM) images (**top** row); Energy Dispersive Spectra (**bottom** row).

Scanning Electron Microscope (SEM) images are displayed in the top row of Figure 3 at two different magnifications. As observed in the images, the carbon particles are generally around 5 μm in size, with some smaller particles measuring down to approximately 0.5 μm. The Energy Dispersive X-ray Spectroscopic (EDS) data are shown in the bottom row of Figure 3. The EDS results indicate that the carbon sample contains elements such as silicon and aluminum. While oxygen is naturally derived from the Alga precursor, the presence of these inorganic elements is likely due to contamination from the porcelain boat used during the chemical activation process. A detailed elemental composition is provided in Table S1 in the Supplementary Materials. According to the table, the activated carbon contains approximately 65.8 at.% carbon, 17.9 at.% oxygen, and about 16.3 at.% of other inorganic impurities.

**Figure 4:**
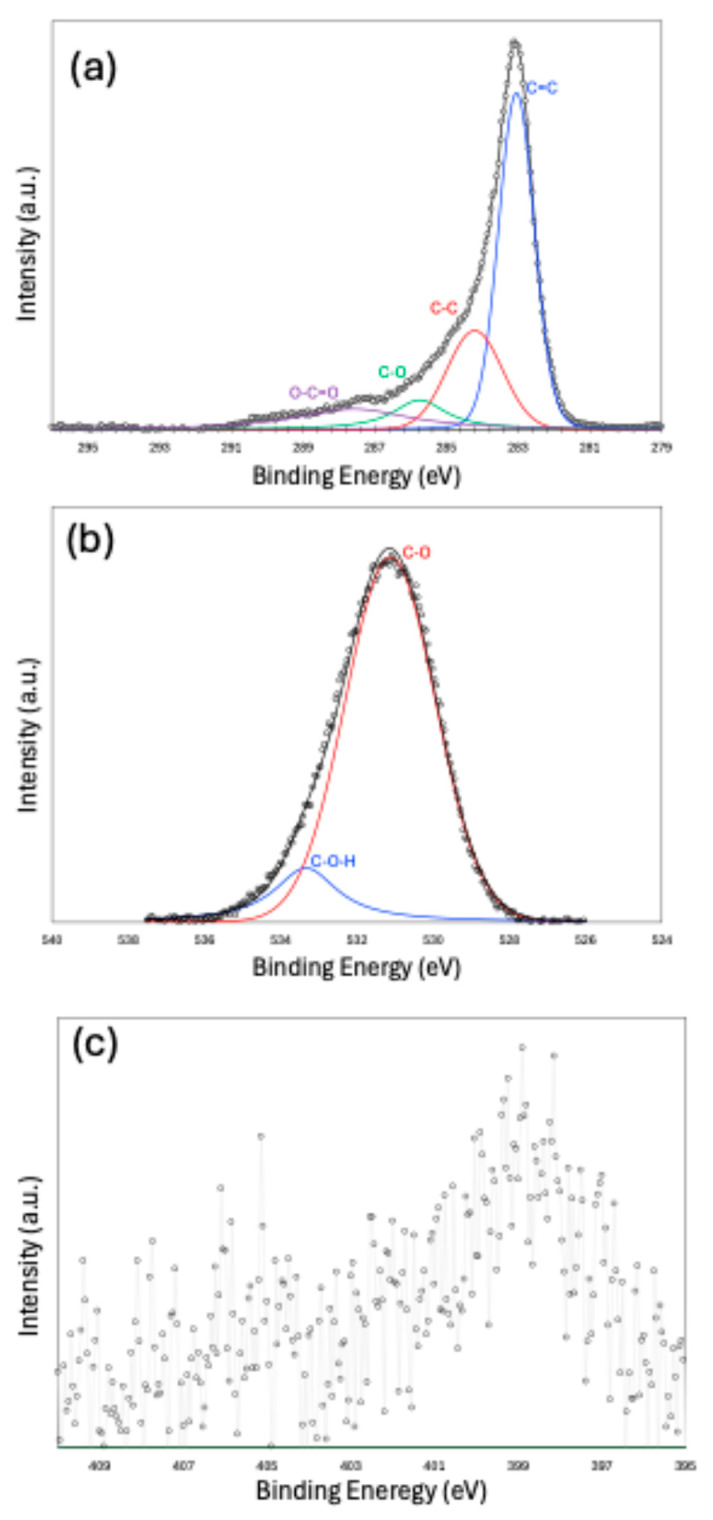
X-ray Photoelectron Spectroscopy (XPS) results for C-1s (**a**), O-1s (**b**), and N-1s (**c**).

X-ray Photoelectron Spectroscopy (XPS) peak fitting results are presented in Figure 4a–c for the C 1s, O 1s, and N 1s peaks, respectively. The C 1s spectrum indicates that the carbon structure is predominantly composed of a sp^2^-hybridized carbon, with a smaller contribution from sp^3^-hybridized carbon. Functional groups, such as C–O and O–C=O, are also present. The peak fitting of the O 1s spectrum confirms the existence of oxygen-containing functionalities. Due to the low nitrogen content in the sample, peak fitting the N 1s spectrum was challenging. However, the fitting suggests that nitrogen is most likely present in the form of pyridinic and nitroso functional groups. Owing to the very small amount of sulfur and noisy data, we did not make an attempt to fit the S-2p spectra.

**Figure 5:**
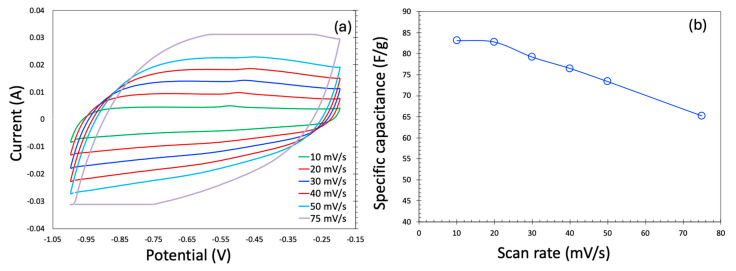
Cyclic voltammetry in the three-electrode system generated in KOH as an electrolyte (**a**) and specific capacitance as a function of scan rate (**b**).

The cyclic voltammetry (CV) profiles for the KOH electrolyte are presented in Figure 5a. At low scan rates, the CV curves exhibit a nearly rectangular shape, indicative of ideal electric double-layer capacitor (EDLC) behavior, which is highly desirable for supercapacitor applications. However, as the scan rate increases, the CV profile becomes noticeably distorted—a trend consistent with previous reports. Ideally, during the supercapacitor operation, the current should remain relatively constant throughout the voltage sweep. At elevated scan rates, however, the voltage changes more rapidly than ions can diffuse into the carbon’s micro- and mesoporous structures. This limits charge storage to the outer surface or larger pores, reducing ionic accessibility and resulting in incomplete charging and lower current responses, thereby distorting the CV shape. Additionally, at high current densities, internal resistance leads to a significant IR (ohmic) drop, which alters the electrode potential and contributes to the skewing of the CV curves, particularly near the potential window limits.

Specific capacitance values (F/g) were calculated from the CV data using the standard expression:

(1) $$C=\frac{A}{2mk∆v}$$

where C = Specific capacitance (F/g);

A = Area of the CV plot (A.V);

m = Mass of active material in the electrode (g);

k = Scan rate (V/s);

∆v = Potential window of voltage sweep (V).

Figure 5b shows the scan rate dependence of specific capacitance for both aqueous and organic electrolytes across the two carbon samples. As expected, the specific capacitance decreases with increasing scan rate, a behavior widely observed in the literature. This decline is primarily due to diffusion limitations. At low scan rates, the slow voltage sweep allows electrolyte ions sufficient time to penetrate the entire pore network of the electrode, thereby maximizing access to active sites and enhancing charge storage. Conversely, at higher scan rates, rapid potential changes restrict ion movement mostly to the electrode surface and larger pores, reducing the effective surface area and thus lowering the specific capacitance.

### 2.2. CDI Experiments

**Figure 6:**
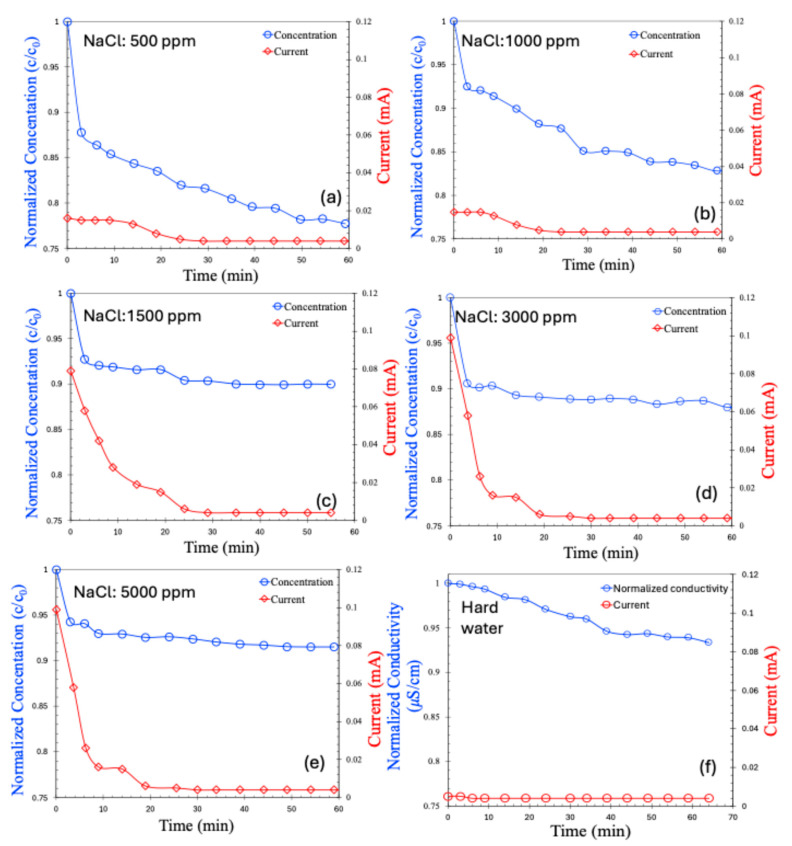
Concentration (normalized) and current (mA) for batch MCDI process in various concentrations of NaCl, 500 ppm (**a**), 1000 ppm (**b**), 1500 ppm (**c**), 3000 ppm (**d**), 5000 ppm (**e**), hard water (**f**).

The CDI results for the batch process are presented in Figure 6a–e for saline water at varying concentrations and in Figure 6f for hard water. As observed, the salt concentration in all cases decreased over time and eventually stabilized at a saturated value. For saline water with low salt concentrations (500–1000 ppm), the decrease in concentration was relatively gradual. In contrast, higher-concentration samples (1500–5000 ppm) exhibited a rapid initial drop in salt concentration, which then plateaued. Correspondingly, the current response across the electrodes followed a similar trend. Notably, the initial current was higher for solutions with greater salt concentrations—an expected outcome, attributable to the higher ionic strength caused by the greater number of charge carriers.

In the case of hard water, both the change in conductivity and the initial current were significantly smaller compared to the saline samples, despite a total salt concentration of approximately 898 ppm. Interestingly, the current generated by hard water was even lower than that for 500 ppm saline water. This can be attributed to several factors:

(1) Stronger hydration shells due to a higher charge density: Divalent ions, such as Ca^2+^ and Mg^2+^ commonly present in hard water have higher charge densities, which result in stronger hydration shells. These shells are energetically difficult to remove, making it more challenging for the ions to pass through ion-exchange membranes and be adsorbed into the electrical double layers (EDLs) of the electrodes.
(2) Lower ionic mobility: Divalent ions exhibit lower mobility in aqueous solutions due to their larger hydrated radii. This reduces their diffusion rate toward the electrodes, thus limiting the efficiency of ion removal.
(3) Electrostatic competition and charge screening: The presence of divalent ions increases the ionic strength of the solution, leading to charge screening effects that can diminish the local electric field near electrode surfaces, thereby reducing ion adsorption efficiency.
(4) Membrane selectivity: In membrane capacitive deionization, the selective transport properties of the membranes may further hinder divalent ion migration due to steric hindrance, thereby reducing their contribution to overall deionization.

**Figure 7:**
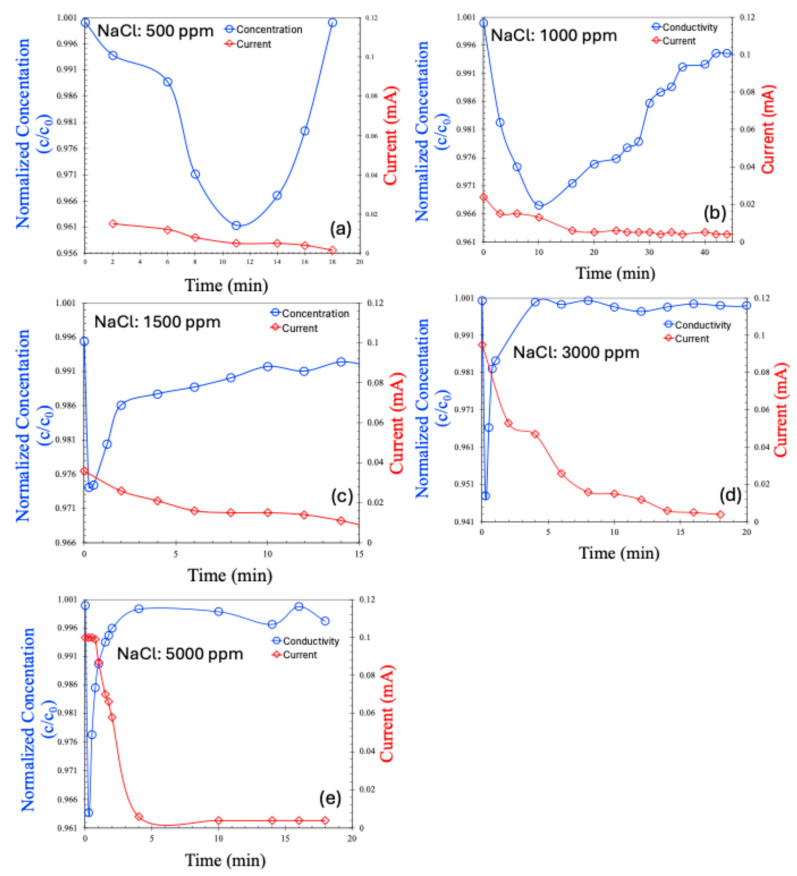
Concentration (normalized) and current (mA) for continuous MCDI process, in various concentrations of NaCl, 500 ppm (**a**), 1000 ppm (**b**), 1500 ppm (**c**), 3000 ppm (**d**), 5000 ppm (**e**).

The CDI results for the continuous process are shown in Figure 7a–e for saline water. Hard water was excluded from the continuous process analysis due to the poor performance observed in the batch mode. In the continuous process, the salt concentration initially decreased, followed by a gradual return to the feed concentration—a trend consistent with previously reported, continuous CDI experiments [22,23,24]. Initially, ions are effectively adsorbed onto the electrodes, leading to a reduction in effluent concentration. However, over time, as the porous electrode surfaces become saturated, they can no longer accommodate additional ions, and the effluent concentration returns to the feed level.

An interesting observation is that the current across the electrodes decreases alongside the initial reduction in salt concentration. However, once electrode saturation is achieved and the salt concentration begins to rise again, the current does not increase accordingly. This is likely due to the saturation of the electrodes; even though the effluent salt concentration matches that of the feed, ion transport through the membranes ceases, preventing any further increase in the current.

It is also noteworthy that the normalized salt concentration in the continuous process is lower than that of the batch process for the same saline water concentrations. This observation is common in membrane capacitive deionization and may be attributed to several factors:

(1) Shorter residence time: In the continuous flow mode, the residence time of water within the cell is shorter, reducing the time available for ions to migrate to the EDLs or pass through the membranes.
(2) Limited control over charge/discharge cycles: The batch process allows for optimized operation with controlled flow and charging/discharging cycles, enhancing ion removal. Continuous flow lacks this level of control, potentially leading to the incomplete regeneration of electrodes.
(3) Flow-induced mass transfer limitations: While flow enhances ion delivery, higher flow rates can lead to boundary layer effects near electrode surfaces that hinder ion transport to adsorption sites. In contrast, the batch process benefits from diffusion-dominated transport, which may be more effective under quiescent conditions.

Although the overall ion removal or salinity reduction in this membrane capacitive deionization process appears modest, it is important to emphasize that only two electrodes were used in the present configuration. In practical applications, CDI systems typically employ multiple electrode stacks, which significantly increase the available surface area and, consequently, improve the salt removal efficiency.

**Figure 8:**
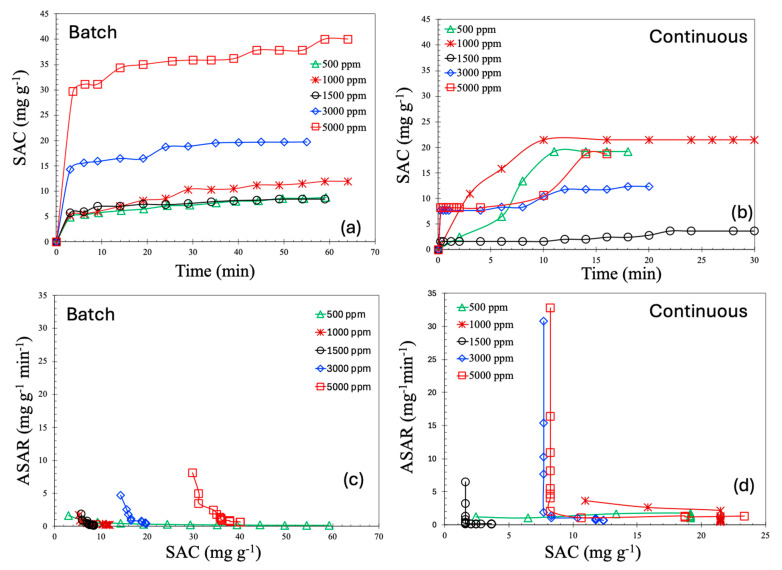
Salt adsorption capacity (SAC) for batch (**a**) and continuous MCDI processes (**b**). Average salt adsorption rate (ASAR) for batch (**c**) and continuous MCDI processes (**d**).

To accurately assess the performance of a deionization process, it is a standard practice to calculate the salt adsorption capacity (SAC, mg/g), which quantifies the amount of salt removed per unit mass of electrode material. The SAC as a function of time for both batch and continuous processes is shown in Figure 8a and b, respectively. As can be observed from these figures, the SAC values for the continuous process are consistently lower than those of the batch process. Moreover, the saturation levels of SAC in this study are comparable to—or in some cases, higher than—values reported in the literature under similar operating conditions. For batch process, the SAC values lie in the range of 30–40 mg/g, whereas for continuous process, they lie at around 20 mg/g. According to the literature, the majority of materials demonstrated SAC values in the range of 5–15 mg/g [12]. A handful of works reported SAC values in the range of 15–20 [25,26,27]; a SAC of 35 mg/g is even more rare [28].

All SAC profiles in both modes of operation exhibit a common behavior: a sharp increase during the initial phase, followed by a steady plateau. This characteristic trend is typical of capacitive deionization (CDI) systems. In the early stage of operation, the concentration gradient between the bulk solution and the electrode surface is steep, creating a high chemical potential that facilitates rapid ion transport toward the electrodes. Simultaneously, the electric field is strongest when the electrodes are relatively unoccupied, further enhancing ion adsorption and resulting in a high SAC during the early time period. As more ions are captured within the electric double layers (EDLs) of the porous electrodes, the number of available adsorption sites decreases, leading to slower uptake. Additionally, electrostatic repulsion between adsorbed and incoming ions increases, further inhibiting additional ion adsorption.

In the case of the batch operation, the ion concentration in the bulk solution gradually decreases over time as ions are removed, leading to a diminishing driving force for ion migration and a corresponding reduction in the SAC. This behavior can be conceptually understood by comparing the CDI system to a conventional capacitor. Initially, the electrodes, acting as capacitive plates, attract ions rapidly due to the applied electric potential. However, as adsorption proceeds and the electrode becomes saturated, the electrostatic potential across the EDLs reaches equilibrium, slowing further ion movement. As a result, the system approaches an asymptotic limit, beyond which the SAC does not significantly increase despite continued operation at the same applied voltage.

An important observation from the experimental results is the non-monotonic trend in the SAC with respect to feed salt concentration. Specifically, both batch and continuous processes show a decline in the SAC when the concentration is increased from 1000 ppm to 1500 ppm, followed by an increase from 1500 ppm to 5000 ppm. This behavior can be explained through the unique ion transport mechanisms involved in membrane capacitive deionization (MCDI). In MCDI, ion-exchange membranes are employed to selectively allow the passage of counter ions while repelling co-ions. At lower salt concentrations, the membrane selectivity is more effective, and the electrodes operate efficiently. As the concentration increases moderately, membrane selectivity decreases, allowing some co-ions to enter the EDLs, which in turn reduces the net salt removal efficiency.

Moreover, at low concentrations, the Donnan exclusion effect becomes significant, increasing the electrostatic barrier for co-ion penetration and causing the EDLs to overlap within the porous structure of the carbon electrodes. As the salt concentration continues to increase, the EDLs become more compressed due to the higher ionic strength of the solution. This compression reduces the effective adsorption volume per unit charge, requiring more electrical energy to adsorb the same amount of salt, which may appear as a drop in the SAC. However, at even higher salt concentrations, the feed solution becomes highly conductive, enhancing the ion transport efficiency under the applied electric field. Increased conductivity improves ion mobility and accelerates adsorption kinetics, allowing more ions to be captured within the same time frame. At these higher concentrations, the surplus of ions in the feedwater makes it easier to fully utilize the electrode capacity, leading to an increase in the measured SAC. Additionally, the thinning of the EDLs due to elevated ionic strength further improves the capacitive behavior of the system by reducing the distance over which the electric field must act. These combined effects explain the observed increase in the SAC at higher salt concentrations and suggest that optimizing feed concentration is crucial for maximizing the performance of MCDI systems.

The average salt adsorption rate (ASAR, mg·g^−1^·min^−1^) is a critical performance metric in capacitive deionization (CDI) processes, as it reflects the rate at which ions are removed from the solution. The ASAR is especially important for scaling, system design, and for balancing economic and energy efficiency considerations. Figure 8c,d present the ASAR as a function of the SAC.

As illustrated in these figures, the ASAR is higher at lower SAC values and decreases rapidly as the SAC increases. This trend mirrors the SAC-time profiles shown in Figure 8a,b, where the SAC increases sharply during the initial phase, followed by a plateau. Notably, the initial ASAR is higher for the continuous process compared to the batch process. In the continuous mode, saline water flows steadily through the MCDI cell, maintaining a consistently high salt concentration at the inlet. This ensures a sustained concentration gradient between the feed solution and the electrode surface, generating a strong driving force for ion transport and resulting in a higher initial ASAR. In contrast, the batch process operates with a fixed volume of saline water. As deionization progresses, the salt concentration in the bulk solution gradually decreases, weakening the driving force for ion adsorption even though the electrode is not yet saturated. Consequently, the ASAR declines more gradually in the batch mode.

Furthermore, the decline in the ASAR with increasing SAC is significantly steeper in the continuous process compared to the batch mode. This is due to the faster saturation of the electrodes under continuous operation, driven by the persistent ion flux. As the electrodes approach their adsorption capacity, the rate of additional ion uptake drops sharply, leading to a pronounced decrease in the ASAR. In the batch mode, however, the system is governed primarily by diffusion, and the gradual reduction in bulk ion concentration leads to a smoother, more progressive decline in the ASAR. Additionally, the continuous mode is characterized by shorter ion residence times within the cell. Once the electrodes near saturation, incoming ions are more likely to pass through the system without being adsorbed, which reduces the observed ASAR despite a continued, albeit slower, increase in the SAC. As a result, the continuous mode reaches its kinetic limitations more rapidly, leading to an abrupt drop in the ASAR. In contrast, the batch mode experiences a more gradual decline, consistent with its diffusion-limited transport dynamics. It is also important to note that the ASAR values reported in the literature [12] are around 0.1 to 10 mg·g^−1^min^−1^, but our reported ASAR values are higher than that.

**Figure 9:**
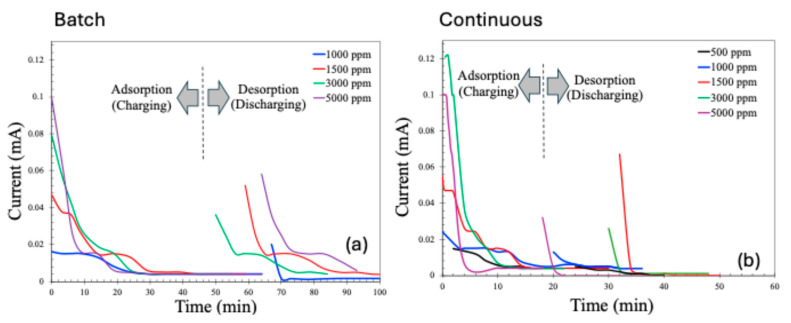
Charging and discharging current patterns for batch (**a**) and continuous (**b**) MCDI processes.

The current profiles during the adsorption (charging) and desorption (discharging or recovery) phases of the CDI process are presented in Figure 9. As expected, the adsorption current decreases over time due to the progressive saturation of the electrodes with adsorbed ions. Similarly, the desorption current is initially high, driven by the rapid release of a large number of previously adsorbed ions upon the reversal of electrode polarity. However, as the desorption process proceeds and the concentration of releasable ions diminishes, the discharge current correspondingly declines.

Specific energy consumption (SEC, kJ/mol) is an important parameter of any CDI process to understand the energy required for the process. SEC is calculated as

(2) $$SEC\;(\frac{kJ}{mol})=\frac{(\int_{0}^{t}V\left(t\right)I\left(t\right)dt)/1000}{{(m}_{salt}/M_{w})}$$

where *V*(*t*) and *I*(*t*) are the time-dependent voltage (v) and current (A), respectively. For constant voltage, *V*(*t*) becomes *V* and is placed outside the integration. $m_{salt}\;$ and $M_{w}\;$are the mass of salt adsorbed during charging and the molecular weight of salt, respectively.

**Figure 10:**
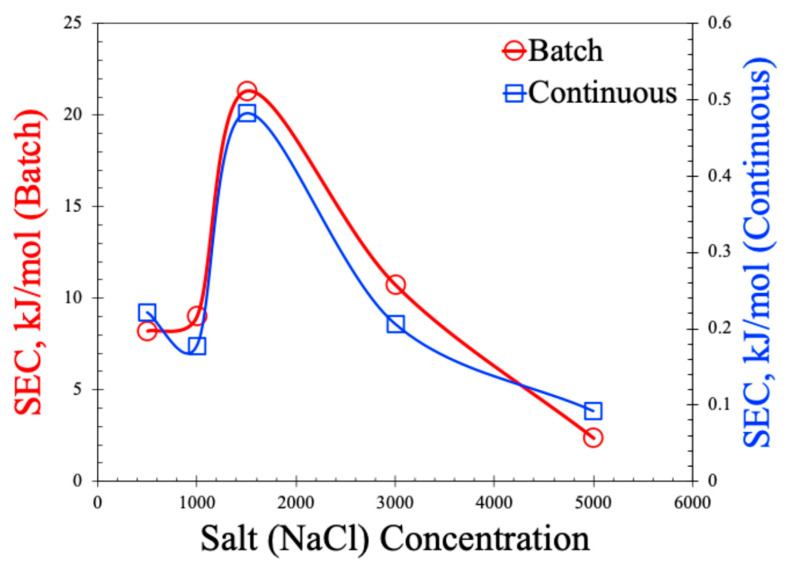
Specific energy consumption (SEC) for batch and continuous MCDI processes.

The SEC values are shown in Figure 10. As observed in this figure, SEC values are two orders of magnitude lower for the continuous process compared to the batch process. The non-monotonic behavior of the SEC with the increase in salt concentration is also noted in the plot. Lower SEC values in the continuous process are expected for the MCDI and primarily attributed to the larger concentration gradient in the continuous process. In the batch mode, the same volume of water is recirculated causing the lowering of the bulk salt concentration, thereby reducing the driving force for ion mitigation. But, as the same voltage is applied and only fewer ions are available to move, it leads to more energy consumption per mole of salt. On the other hand, in the continuous MDI, high-salinity water is constantly fed to the cell, which keeps the concentration gradient high, allowing for faster and more efficient ion removal leading to the adsorption of more salt per unit of energy input. In addition, the batch process often requires longer absorption times and the latter stages of the cycle consume nearly the same amount of power but yield very little salt removal, inflating the SEC. The non-monotonic behavior of SEC with respect to the salt concentration resonates with the non-monotonic behavior of the SAC. As observed in Figure 8a,b, the SAC values dropped at 1500 ppm of salt concertation, which was manifested as an increase in SEC values in Figure 10. The underlying reasons of such behavior are the same as explained earlier, and attributed to membrane selectivity, the Donnan exclusion effect, and a variation in the thickness of the electrical double layer (EDL). It is also important to note that the SECs in this work are much lower than those reported in the literature [12,29,30,31,32,33], suggesting our process can be operated at a lower energy.

## 3. Experimental Procedure

### 3.1. Synthesis of Activated Carbon from Alga

The synthesis of activated carbon from Spirulina Alga was carried out in multiple steps. Initially, the Alga was subjected to hydrothermal carbonization. This was performed by heating the 17.5 g commercially available Alga in 55 mL of water inside a sealed hydrothermal reactor at 250 °C for 24 h. The resulting hydrochar sludge was recovered upon cooling and subsequently washed with deionized water to remove soluble impurities. The sludge was dried, loaded to a porcelain boat, and inserted in a Lindberg-Blue^TM^ tube furnace (Thermo-Fisher Scientific, Waltham, MA, USA). The furnace was heated to 800 °C at the ramp rate of 10 °C/min followed by cooling under a nitrogen atmosphere. In order to activate the carbon, the carbonized mass was mixed with KOH in a 1:4 ratio and loaded in the same tube furnace. The furnace was further heated to 1000 °C at the ramp rate of 10 °C/min and cooled down under a nitrogen atmosphere. Finally, the activated carbon mask was washed with DI water several times followed by filtering and drying.

### 3.2. Characterization of Activated Carbon

The activated carbon was characterized by pore textual properties, Scanning Electron Microscopic Imaging-Energy Dispersive X-ray Spectroscopy (SEM-EDS), and X-ray Photoelectron Spectroscopy (XPS). The SEM-EDS data were obtained with a FEI Quanta 600 FEG Mark II Environmental Scanning Electron Microscope (ESEM) (Thermo Fisher Scientistic, Waltham, MA, USA). The X-ray Photoelectron Spectroscopy (XPS) measurements were performed with a Thermo K-alpha instrument (Thermo Fisher Scientistic, Waltham, MA, USA) with a monochromated Al Kα Source (1486.7 eV) and overall 0.7 eV resolution. The charge compensation was performed by using low-energy electrons and ions. The potential properties, including BET surface area and pore size distribution, were calculated by measuring N_2_ adsorption–desorption at 77 K and CO_2_ absorption at 273 K in an Autosorb-Any gas surface area and porosity analyzer (Quantachrome, Boynton beach, FL, USA).

Electrochemical measurements were conducted using a BASi PalmSens4 potentiostat (Palmsense, Houten, The Netherlands). A three-electrode setup was utilized for cyclic voltammetry, with a carbon-coated small electrode acting as the working electrode, an Ag/AgCl electrode as the reference, and a platinum wire as the counter electrode. Cyclic voltammetry was performed at scan rates of 10, 20, 30, 40, 50, and 75 mV/s.

### 3.3. Fabrication of CDI Electrode Compacting CDI Cell

In order to fabricate the electrode, at first, the carbon sample was ball-milled for 20 min. Then, a carbon slurry was prepared by homogenizing the ball-milled activated carbon, super P conductive carbon, and 5% polyvinylidene fluoride (PDF) in dimethyl acetamide (DMAc) with a mortar and pestle, followed by diluting the slurry by acetone. After that, the slurry was applied on 7 cm × 7 cm commercial graphite foil by the drop casting method. Finally, the two graphite electrodes were finished by drying them in an oven at 100 °C overnight.

**Figure 11:**
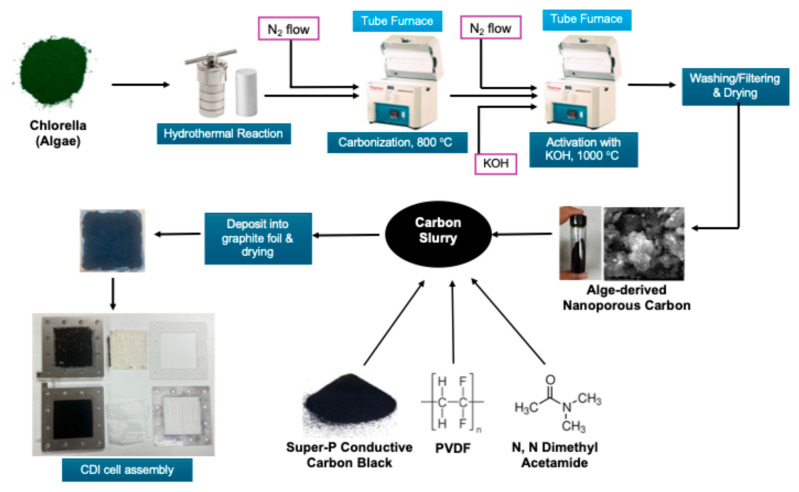
Schematic of fabrication of CDI electrodes.

The CDI cell was compiled by putting 2 electrodes in a commercially available cell with an active electrode area of 7 cm × 7 cm. A properly sized and commercially available cation exchange membrane and anion exchange membrane were inserted near the negative and positive electrodes of the CDI cell, respectively. The schematic of the entire process, including carbon synthesis to CDI cell formation, is shown in Figure 11.

### 3.4. CDI Experiments with Saline and Hard Water

**Figure 12:**
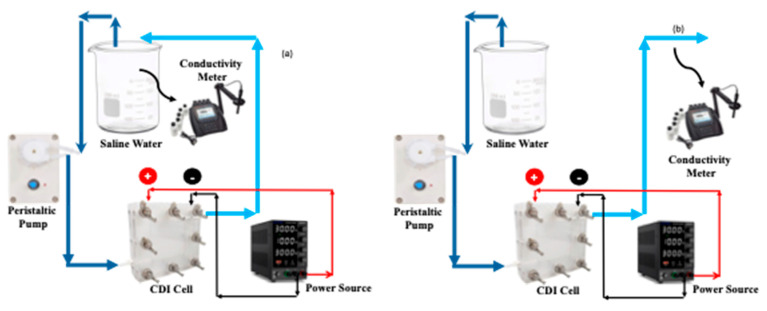
Schematic of batch (**a**) and continuous (**b**) MCDI processes.

For all CDI experiments, 1.50 V of potential was applied between the positive and negative terminals of the CDI cell. For saline water samples, the concentrations that were employed were 500, 1000, 1500, 3000, and 5000 ppm. The hard water sample was made by mixing 288 ppm of CaSO_4_, 220 ppm of MgSO_4_, and 390 ppm of NaHCO_3_. The entire operation of CDI was performed using two different modes: batch mode or closed loop and continuous mode or open loop. This schematic of the batch and continuous processes are shown in Figure 12. The concentration of the saline and hard water was measured by an Orion star conductivity meter (Thermo Fisher Scientistic, Waltham, MA, USA). For saline water, the concentration was calibrated against the true concentration, however the calibration was not performed for hard water owing to the presence of valence ions. In each mode and at each concentration, once the exit concentration became the same as that of the feed water, the polarity of the electric potential was switched to desorb the salt ions followed by restarting the adsorption process upon switching back the electric potential. At every time interval, when the conductivity of effluent water was measured, the current between the electrodes was also recorded using the same variable power source.

## 4. Conclusions

This study presents a sustainable and effective approach to producing high-performance activated carbon from Spirulina Alga through hydrothermal carbonization followed by KOH activation. The resulting material exhibited a high BET surface area (1747 m^2^/g), hierarchical porosity, and favorable surface chemistry, as confirmed by SEM-EDS, XPS, and gas adsorption analyses. When applied in membrane capacitive deionization (MCDI), the electrodes achieved promising salt adsorption capacities (SACs) ranging from 25 mg/g in the batch mode to 40 mg/g in continuous flow, with rapid ion removal characterized by high average salt adsorption rates (ASARs) exceeding 10–30 mg·g^−1^·min^−1^ in the initial phase of operation. While the SAC and ASAR profiles confirmed standard CDI behavior—rapid initial uptake followed by saturation—the non-linear relationship between the feed concentration and SAC suggests complex interactions between ion transport dynamics, electric double-layer compression, and membrane ion selectivity. Particularly, the poor performance with hard water underlines the challenge posed by divalent ions, reinforcing the importance of tailoring membrane and electrode properties for such cases. Despite the two-electrode limitation and absence of long-term cycling tests, the system maintained a consistent performance during repeated short-term runs and exhibited low specific energy consumption (SEC), in the range of 8–21 kJ/mol in the batch mode and 0.1–0.5 kJ/mol in continuous operation. These metrics are comparable to or surpass those of other single-electrode CDI systems reported in the recent literature. Overall, the results demonstrate the strong potential of Alga-derived activated carbon as an eco-friendly and efficient electrode material for next-generation water desalination technologies, while also highlighting the need for continued investigations into long-term performance and ion-specific design strategies.

## Data Availability

Data are contained within the article or Supplementary Material.

## Supplementary Materials

The following supporting information can be downloaded at: https://www.mdpi.com/article/10.3390/molecules30132848/s1, Table S1: Constituents of the carbon produced from Alga.
