# Supplementary material for: Nanoporous Carbons from Hydrothermally Treated Alga: Role in Batch and Continuous Capacitive Deionization (CDI)
Source: Molecules. 2025 Jul 3;30(13):2848. doi: 10.3390/molecules30132848 (PMC12250988; doi:10.3390/molecules30132848)
Supplement: Supplementary file 1 [file molecules-30-02848-s001.zip › molecules-3682882-supplementary.pdf]

Supplementary Information

Nanoporous Carbons from Hydrothermally Treated Alga: Role in Batch and Continuous Capacitive Deionization (CDI)

Dipendu Saha*, Ryan Schlosser, Lindsay Lapointe, Marisa L. Comroe, John Samohod, Elijah Whiting and David S. Young

Chemical and Materials Engineering Department,

Widener University,

1 University Place, Chester PA, 19013, USA

Table S1. Constituents of the carbon produced from Alga

**Elements**	**Atom%**	**Wt.%**
C	65.8	50.4
O	17.9	18.3
Others (Si, Ai, Ca, Mg etc.)	16.3	31.3
